# Global Expansion of Linezolid-Resistant Coagulase-Negative Staphylococci

**DOI:** 10.3389/fmicb.2021.661798

**Published:** 2021-09-13

**Authors:** Vladimir Gostev, Semen Leyn, Alexander Kruglov, Daria Likholetova, Olga Kalinogorskaya, Marina Baykina, Natalia Dmitrieva, Zlata Grigorievskaya, Tatiana Priputnevich, Lyudmila Lyubasovskaya, Alexey Gordeev, Sergey Sidorenko

**Affiliations:** ^1^Department of Medical Microbiology and Molecular Epidemiology, Pediatric Research and Clinical Center for Infectious Diseases, Saint Petersburg, Russia; ^2^Department of Medical Microbiology, North-Western State Medical University Named After I. I. Mechnikov, Saint Petersburg, Russia; ^3^Infectious and Inflammatory Disease Center, Sanford Burnham Prebys Medical Discovery Institute, La Jolla, CA, United States; ^4^Laboratory of Clinical Microbiology, National Agency for Clinical Pharmacology and Pharmacy, Moscow, Russia; ^5^Saint Petersburg State University, Saint Petersburg, Russia; ^6^Department of Microbiology, N. N. Blokhin Russian Cancer Research Center, Moscow, Russia; ^7^Department of Microbiology, Clinical Pharmacology and Epidemiology, National Medical Research Center for Obstetrics, Gynecology and Perinatology, Moscow, Russia

**Keywords:** coagulase-negative staphylococci, linezolid, resistance, genome epidemiology, tedizolid

## Abstract

Coagulase-negative staphylococci (CoNS) for a long time were considered avirulent constituents of the human and warm-blooded animal microbiota. However, at present, *S. epidermidis*, *S. haemolyticus*, and *S. hominis* are recognized as opportunistic pathogens. Although linezolid is not registered for the treatment of CoNS infections, it is widely used off-label, promoting emergence of resistance. Bioinformatic analysis based on maximum-likelihood phylogeny and Bayesian clustering of the CoNS genomes obtained in the current study and downloaded from public databases revealed the existence of international linezolid-resistant lineages, each of which probably had a common predecessor. Linezolid-resistant *S. epidermidis* sequence-type (ST) 2 from Russia, France, and Germany formed a compact group of closely related genomes with a median pairwise single nucleotide polymorphism (SNP) difference of fewer than 53 SNPs, and a common ancestor of this lineage appeared in 1998 (1986–2006) before introduction of linezolid in practice. Another compact group of linezolid-resistant *S. epidermidis* was represented by ST22 isolates from France and Russia with a median pairwise SNP difference of 40; a common ancestor of this lineage appeared in 2011 (2008–2013). Linezolid-resistant *S. hominis* ST2 from Russia, Germany, and Brazil also formed a group with a high-level genome identity with median 25.5 core-SNP differences; the appearance of the common progenitor dates to 2003 (1996–2012). Linezolid-resistant *S. hominis* isolates from Russia demonstrated associated resistance to teicoplanin. Analysis of a midpoint-rooted phylogenetic tree of the group confirmed the genetic proximity of Russian and German isolates; Brazilian isolates were phylogenetically distant. *repUS5*-like plasmids harboring *cfr* were detected in *S. hominis* and *S. haemolyticus*.

## Introduction

Coagulase production was introduced as a criterion for the differentiation of members of genus *Staphylococcus* members in 1940 (Fairbrother, 1940). In contrast to the main representative of coagulase-positive staphylococci (*Staphylococcus aureus*), coagulase-negative staphylococci (CoNS) initially were considered avirulent constituents of the human and warm-blooded animal microbiota. However, at present, many CoNS species are recognized as opportunistic pathogens (Coates et al., 2014; Heilmann et al., 2019). The most frequent colonizers of human skin *S. epidermidis, S. haemolyticus*, and *S. hominis* are the main cause of local and bloodstream foreign body–related infections; prosthetic valve endocarditis (Otto, 2012; Becker et al., 2014); and neonatal infections, including bacteremia (Dong and Speer, 2014).

Treatment of CoNS is becoming increasingly complex due to the emergence and rapid spread of methicillin resistance (MR)—a marker of resistance to most beta-lactams (except for ceftaroline and ceftobiprol), mediated by an additional penicillin-binding protein (PBP), designated PBP2a, that has reduced affinity to beta-lactams. After its first description (Kjellander et al., 1963), prevalence of MR among CoNS causing hospital-acquired infections has continuously increased. Publications from the late 2010s confirm high percentages of MR isolates among CoNS causing bacteremia worldwide: 64.2% in the United Kingdom (Henriksen et al., 2018), 64.7% in the United States (Pfaller et al., 2019), and 91% in Iran (Pourakbari et al., 2018).

Methicillin resistance in CoNS is frequently associated with resistance to other antibiotics except for glycopeptides, which for many years were the drugs of choice in the treatment of staphylococcal infections. Over the past decades, treatment options for Gram-positive infections have expanded significantly with new glycopeptides, beta-lactams, lipopetides, glycylcyclins, and oxazolidinones (linezolid and tedizolid). Although linezolid is not registered for the treatment of CoNS infections, it was used off-label for the treatment of meningitis (Krueger et al., 2004; Kruse et al., 2006; Watanabe et al., 2013), ventriculitis (Boak et al., 2006), osteomyelitis (Nam et al., 2008) and prosthetic-joint infections (Ferry et al., 2018) caused by CoNS. However, high rates of oxazolidinone consumption or the use of long courses of therapy promotes resistance (Dortet et al., 2018; Bai et al., 2019). There are four mechanisms of oxazolidinone resistance in CoNS: methylation of 23S rRNA [plasmid-born chloramphenicol–florfenicol resistance (*cfr)* gene], mutations in 23S rRNA and ribosomal proteins (*rpl* genes), and efflux (plasmid-born *optr*A gene) (Long and Vester, 2012; Wang et al., 2015). Resistance due to ribosomal protection (plasmid-born *poxt*A gene) was recently described in enterococci (Antonelli et al., 2018). Isolates harboring the *cfr* gene are resistant to linezolid but susceptible to tedizolid; all other resistance mechanisms confer cross-resistance between both oxazolidinones. Oxazolidinone-resistant CoNS infections and particularly bloodstream infections are associated with poor clinical outcome: high mortality and prolonged hospital stay (Russo et al., 2015).

Recently, several outbreaks of hospital-acquired infections due to oxazolidinone-resistant CoNS were reported from the United States (Tewhey et al., 2014), Brazil (de Almeida et al., 2013), Greece (Karavasilis et al., 2015), Italy (Mendes et al., 2010), France (Dortet et al., 2018), Germany (Layer et al., 2018), China (Cai et al., 2012), and Spain (Seral et al., 2011; Rodríguez-Lucas et al., 2020).

Revealing the genetic structure of bacterial populations is necessary for the identification of their evolution and distribution. Clustering in large databases is most often done using both non-spatial and spatial Bayesian analysis of population structure (BAPS) algorithms developed by Corander et al. (2008). Bayesian evolutionary analysis by sampling trees (BEAST) is used for the estimation of the time of clade formation (or divergence times). It is used to build rooted, time-measured phylogenies inferred using strict or relaxed molecular clock models. Using the combination of BEAST and BAPS (Castillo-Ramirez et al., 2012) several genetically isolated lineages within the MRSA sequence type (ST) 239 clone and chronology of the introduction of these lineages into the specific geographical regions were identified.

In the present study, we describe linezolid-resistant *S. epidermidis* (LRSE), *S. haemolyticus*, and *S. hominis* recovered in several tertiary hospitals in Moscow. Methods of comparative genomics were initially used for the investigation of recovered isolates and followed by comparison with publicly available genomes of oxazolidinone-resistant CoNS. BAPS and chronogram reconstruction using BEAST were implemented to determine clusters and the time of linezolid-resistant CoNS lineage emergence. The possibilities of two scenarios of oxazolidinone resistance dissemination were evaluated: either clonal spread of resistant genetic lineages or emergence of resistance *de novo*.

## Materials and Methods

### Bacterial Strains and Antibiotic Susceptibility

*Staphylococcus epidermidis*, *S. hominis*, and *S. haemolyticus* isolates (*n* = 47) demonstrating reduced susceptibility to linezolid were collected in 2014–2018 in six Moscow hospital laboratories and transferred to the central laboratory Pediatric Research and Clinical Centre for Infectious Diseases (PRCCID) together with record forms. Personal data of patients were not included in record forms; ethical approval for the study was not required. Control of CoNS identification was performed in the central laboratory by MALDI-TOF mass spectrometry (Microflex LT, Bruker Daltonics, Germany) following the manufacturer’s instructions. Antimicrobial susceptibilities to 22 antibiotics (Molekula, United Kingdom), including linezolid (Sigma-Aldrich, United States), tedizolid (Bayer, Germany), teicoplanin, oritavancin, telavancin, and dalbavancin (Biosynth Carbosynth, United Kingdom) were tested by broth microdilution in cation-adjusted Mueller–Hinton broth (Bio-Rad, Marnes-la-Coquette, France) and interpreted according to The European Committee on Antimicrobial Susceptibility Testing (EUCAST) (2020) recommendations (Breakpoint tables for interpretation of MICs and zone diameters, Version 10.0, 2020.^1^).

### Population Analysis Profile

A population analysis profile (PAP) was performed according to the microdilution modification proposed in a previous study (Pfeltz et al., 2001). Four dilutions (10^–1^, 10^–3^, 10^–5^, and 10^–7^) of the initial suspension (10^8^ CFU/mL) of each strain were prepared. Three 10-μL droplets of each dilution were plated on vancomycin-containing brain–heart infusion agar plates (0.5, 1, 1.5, 2.0, 3.0, 4.0, 6.0, 4.0, 8.0, 12.0, and 16.0 mg/mL). The inoculated plates were incubated for 48 h at 37°C. Plated droplets containing 5–50 CFU were selected for counting, and the average number of colonies per vancomycin concentration was determined. Plots showing the log10 CFU in the presence of each concentration of vancomycin were constructed. Area under curve (AUC) was calculated using the R 3.6.3 base package with trapezoidal rule. The ratio of AUC for CoNS to AUC of control hetero-resistant strain *Staphylococcus aureus* Mu50 under the study was calculated (AUC_CoNS_/AUC_Mu50_). Isolates demonstrating AUC_CoNS_/AUC_Mu50_ ≥ 0.9 were considered to be hetero-resistant.

### Whole Genome Sequencing

Genomic DNA was extracted using a PureLink^TM^ Genomic DNA Mini Kit (Invitrogen^TM^, CA, United States) with preliminary lysis of the cells being done with 1 mg/mL lysostaphin (Sigma-Aldrich, United States). The Nextera XT Kit (Illumina, San Diego, CA, United States) was used for DNA library preparation followed by sample indexing and amplification according to the manufacturer’s protocol. DNA libraries were sequenced on a MiSeq instrument (Illumina, San Diego, CA, United States). Quality check data on sequencing reads is presented in Supplementary Table 1.

### Genome Assembling and Annotation

Sequence reads were filtered and trimmed using the trimmomatic 0.32 (Bolger et al., 2014) under default settings for Illumina raw data. Read quality and length distribution were analyzed with FastQC 0.11.9 (Brown et al., 2017). *De novo* contigs were assembled with SPAdes 3.14.0 (Bankevich et al., 2012). Bowtie 2 2.3.5 (Langmead and Salzberg, 2012) and SAMtools 1.10 (Li et al., 2009) software were used for detection of SNP‘s conferred antibiotic resistance, including oxazolidinones resistance. Prediction of single nucleotide polymorphism (SNP) effects was done with SNPeff 4.3t (Cingolani et al., 2012) using filtered (minimum SNP coverage 10 and quality Phred per base > 20) and deduplicated reads after SAMtools processing. The following genomes were used as reference: *S. epidermidis* ATCC 12228 (NC_004461.1), *S. hominis* FDAARGOS_746 (NZ_CP046306.1), and *S. haemolyticus* ATCC 29970 (NZ_CP035291.1). Genomes were annotated with PROKKA 1.14.5 (Seemann, 2014), MLST typing, resistance, and virulence gene typing were done using MLST 2.18.0^2^ and abricate 0.9.8^3^ scripts, respectively.

### Inclusion of Genome Data From Previous Studies

For phylogenetic reconstruction in addition to Moscow genomes, data from previous studies—outbreaks in France (Dortet et al., 2018) and the United States (Tewhey et al., 2014)—were included in the study. These data were downloaded from the NCBI Sequence Read Archive (SRA) (BioProjects PRJEB22222 and PRJNA239883, respectively), assembled, and annotated using methods listed in the previous section. Additionally, 460 genomes of *S. epidermidis*, 60 genomes of *S. hominis*, and 205 genomes of *S. haemolyticus* were downloaded from the NCBI GenBank using the list of genomes from the PATRIC database (update July 2019) (Wattam et al., 2017). The genomes included for phylogenetic analysis are listed in the data set (Supplementary Table 2).

### Phylogenetic and Pan-Genome Analysis

Pan-genomic analysis was done with Roary 3.13.0 (Page et al., 2015), and gene content comparison was done with scoary 1.6.16 (Brynildsrud et al., 2016). Genomes of the CoNS were *in silico* genotyped against the PubMLST database update July 2020 (Jolley and Maiden, 2010) using MLST script 2.18.0 (see text footnote 2).

To produce a core genome alignment for phylogenetic tree reconstruction, we developed a nucmer aligner wrapper named panmap (available on^4^). Panmap uses nucmer 3.9.4 (Kurtz et al., 2004) to create a pairwise alignment for every genome against a reference contigs [in our case, we used the complete chromosome of *S. epidermidis* BPH0662 (NZ_LT571449.1), of *S. hominis* FDAARGOS_136 (NZ_CP014107.1), and *S. haemolyticus* JCSC1435 (NC_007168.1)]. Then, it uses reference contig annotations for every region—gene or intergenic—to produce counts of gapped positions. A gapped position is defined as a position in which the proportion of gaps is above some threshold. If the proportions of gapped positions in a region are higher than a second threshold, then the whole region is dropped. Otherwise, the whole region is kept. Both thresholds were set to 1%. We implemented this annotation-based region-to-region approach to keep as much information about distance between SNPs as possible as Gubbins 2.4.1 (Croucher et al., 2015)—the program that identifies potential recombination regions—uses SNP density information. Using Gubbins 2.4.1, we removed potential regions of recombination from the core genome alignment. The resulting alignment was used for phylogenetic tree reconstruction by IQ-tree 1.6.12 software with ModelFinder and ultrafast bootstraps (Nguyen et al., 2015; Kalyaanamoorthy et al., 2017; Hoang et al., 2018). The substitution model chosen by ModelFinder was TVMe + ASC + R4. The substitution model was chosen based on the ModelFinder results under default parameters. Long branches that did not contain genomes of interest were removed from the trees. The core genome alignment was clustered using BAPS with the rhierBAPS R package 1.0.1 (Cheng et al., 2013; Tonkin-Hill et al., 2018) with the expected number of populations set as 20 and maximum depth of clustering set as two. Intra- and inter-group pairwise comparison of the number of SNPs was carried out using R script pairwise_snp_differences^5^ (Supplementary Table 3).

### Timed Phylogeny Analysis

Timed phylogeny calculation was used for the genetically closest groups of CoNS. Several genome groups were chosen: LRSE belonging to ST2 (*n* = 76), all (susceptible and resistance to linezolid) ST22 isolates (*n* = 27), and *S. hominis* ST2 (*n* = 20). BEAST 2.6.4 was used to generate a timed phylogeny (chronogram) assuming a relaxed lognormal clock and, with coalescent constant tree prior, 10 million iterations of a gamma site model with an HKY substitution model. Tree convergence was confirmed using BEAST’s Tracer 1.7.1 program (Suchard et al., 2018) using the recommended criterion (ESS > 200). TreeAnnotator was then used to identify the maximum clade credibility (MCC) tree using a 10% burn-in. The resulting tree was visualized using FigTree 1.4.4.

### Annotation and Mapping of Resistance Data

Analysis of resistance-related SNPs was done for SNPs known to cause linezolid resistance: 23S rRNA (G2576T, C2534T, T2504A *Escherichia coli* numbering), ribosomal proteins L3 (Ala157Arg, Asp159Tyr, Met156Thr, Gly152Asp, His146Arg, Gly137Val, Leu101Val), L4 (Asn158Ser), and L2 (Val112Ile, Ile75Thr) with a potential role of oxazolidinone-resistant. *mecA*, *cfr* genes, and mutations in *rpoB* that conferred multidrug resistance were also mapped. The sequence of 23S rRNA was extracted using barrnap tool 0.9^6^. Other genes were extracted with designed in the study Riddikulus script^7^. Genes were aligned by MAFFT 7.407 (Katoh and Standley, 2013). SNPs were extracted using Unipro UGENE software 37.0 (Okonechnikov et al., 2012). Calculation of the total number of acquired resistance genes for each genome was done with abricate 0.9.8 (see text footnote 3).

Seventeen proteins associated with decreased glycopeptide susceptibility in *S. aureus* (MprF, Pbp123, WalKR, GraSR, VraSRT, RpoBC, YycIH, Cmk, and MsrR) were selected for analysis, and homology proteins were extracted from *S. epidermidis* and *S. hominis* genomes. Frequency of amino acids substitution (AAS) was compared across the all-genome data set in linezolid-resistant genomes and linezolid-susceptible genomes. AAS with frequency below the threshold of 5 and 1% for *S. epidermidis* and *S. hominis*, respectively, were excluded. For possible associations between mutations and linezolid-resistance phenotype multiple correspondence analysis (MCA) was applied using factoextra R package 1.0.7.

Visualization and annotations of phylogenetic trees were done using ITOL 6.1.1 (Letunic and Bork, 2016). Plasmid structural comparison was done with Mauve 2.4.0 (Darling et al., 2004).

### Accession Numbers

Genomic data have been deposited in NCBI Sequence Read Archive (SRA) and all reads are available from BioProject PRJNA384130 (SRA id: SRR5482186—SRR5482205 and SRR8427123—SRR8427149).

## Results

### Linezolid-Resistant CoNS in Moscow Hospitals

The first two LRSE isolates were recovered at site A in 2014 and 2015 from patients with catheter-associated bloodstream infections in the intensive care unit. These isolates belonged to genetic lineage ST23. Emergence and dissemination of LRSE (ST2, *ST22*), linezolid-resistant *S. hominis* (ST2), and *S. haemolyticus* (ST1) were observed in several Moscow hospitals (A to F), in 2016–2018.

Different combinations of mutations in 23S rRNA and *rlp3* genes and acquisition of the *cfr* gene mediated resistance to oxazolidinones (Table 1). To estimate the number of modified copies of 23S rRNA, we aligned sequence reads on the target fragment of the reference sequence, and in all isolates, the specific SNPs were detected in 99% of the reads without mixed alleles. These data suggest that mutations are present in all copies of 23S rRNA.

**TABLE 1:** Characterization of Oxazolidinone-resistant CoNS isolated in Moscow hospitals.

**Species**	**MLST**	**N**	**Sites**	**Source**	**MIC, mg/L**		***cfr***	**SNPs**	
					**LNZ**	**TDZ**		23S rRNA*	***rpl3***
*S. epidermidis*	ST2	6	E	Blood, sputum, intubation tube	>32	2–4	−	G2576T, (G2602T)	–
		2	F	Intubation tube, feces	>32	16	−	G2576T, (G2602T)	Gly137Val, His146Arg, Met156Thr
	ST23	2	A	Blood	32	2	−	G2576T, (G2602T)	–
	ST22	20	A, B, D, E	Blood	>32	>32	−	C2534T, (C2560T), T2504A, (T2530A)	Asp159Tyr, Gly152Asp
*S. hominis*	ST2	10	A,C,D,E	Blood	>32	4–8	–	G2576T, (G2603T)	Met156Thr, Val154Leu
		6	A,F	Blood, feces, sputum	>32	4–8	+	G2576T, (G2603T)	Met156Thr, Val154Leu
*S. haemolyticus*	ST1	1	A	Blood	>32	0.25	+	–	–

*Mutation in 23S rRNA according to *E. coli* numbering, in brackets mutation according to Staphylococcus numbering (NC_004461.1, NZ_CP046306.1, and NZ_CP035291.1).

All CoNS isolates demonstrated a high level of linezolid resistance (MIC ≥ 32 mg/L). The majority of isolates demonstrated tedizolid MIC ≤ 16.0 mg/L. A high level of tedizolid resistance (MIC ≥ 32 mg/L) was detected in ST22 isolates carrying double substitution in 23S rRNA. Only one *S. haemolyticus* isolate carrying the *cfr* gene as a single mechanism of resistance demonstrated susceptibility to tedizolid (MIC = 0.25 mg/L).

LRSE, belonging to ST2 and ST22, harbored *mec*–cassette of *SCCmec* III–like type with intact recombinase genes *ccrA3*, *ccrB3*, *mec*-complex class A, and *psm-mecA* regions. ST23 isolates carried *SCCmec* V–like type without the *psm-mecA* region. All *S. hominis* harbored intact SCC*mec* III with *psm-mecA* region. The *S. haemolyticus* isolate lacked SCC*mec* elements with only the *mecA* gene.

CoNS isolates under the study demonstrated high levels of associated resistance to aminoglycosides, fluoroquinolones, macrolides/lincosamides, tetracycline, co-trimoxazole, fusidic acid, rifampicin, and mupirocin but retained susceptibility to ceftaroline, tigecycline, and daptomycin. Resistance phenotypes were confirmed by the detection of corresponding genotypes (Supplementary Table 4). Isolates belonging to ST22 demonstrated susceptibility to erythromycin (despite the presence of intact macrolide resistance genes *msrA* and *mphC*) and resistance to clindamycin (L-phenotype). The phenotype is associated with T2504A point mutation (Liakopoulos et al., 2009).

### Molecular Epidemiology of LRSE

Analysis of the *S. epidermidis* population identified a pan-genome consisting of 31,036 genes and 731 core ortholog gene clusters. Phylogenetic analysis of genomes was based on extraction of a 74,628 nt long core genome after alignment.

**FIGURE 1:**
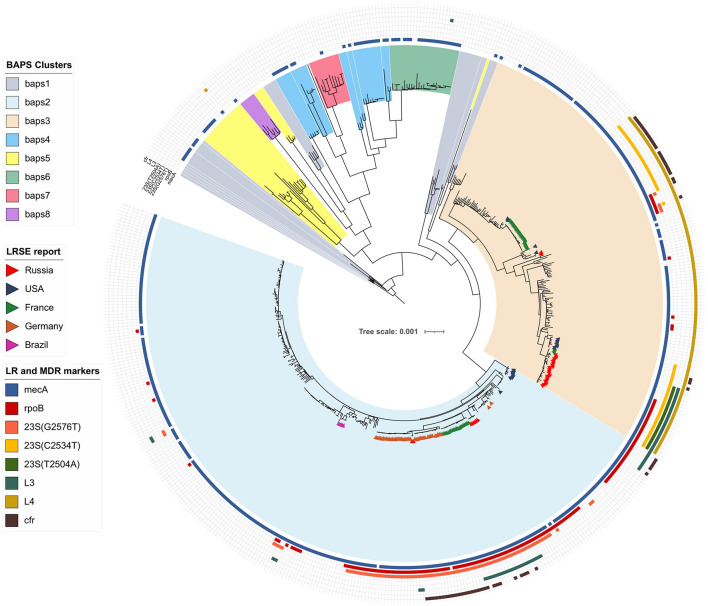
Maximum-likelihood phylogeny of *S. epidermidis* population (*n* = 554) with mapped data of linezolid resistance and multidrug resistance data. Background color fill is matched to BAPS clustering. LRSE isolate is marked triangles: from Russia (current study), the United States (Tewhey et al., 2014), France (Dortet et al., 2018), Germany (unpublished data, only metadata is available BioProject PRJNA314440), and Brazil (unpublished data, only metadata is available from BioProjects PRJNA419710, PRJNA419705, PRJNA419706, and PRJNA419711). Annotation from inner to outer circle: presence *mecA*, mutations in *rpoB* (rifampin resistance), linezolid resistance (mutations in 23S rRNA, *rpl3, rpl4* genes and presence of *cfr*). Full zoom scalable view of phylogenetic tree with additional data and names of strains and description of calculated acquired resistance genes are available in Supplementary Figure 1.

Bayesian analysis of population structure divided the *S. epidermidis* population into eight clusters (Figure 1 and Supplementary Figure 1), but LRSE genomes were found only in two of them: BAPS clusters 2 and 3 consisting mainly of *mecA*-positive isolates of human origin (from infected persons and carriers). *S. epidermidis* belonging to other clusters (1 and 4–8) were isolated from different sources: environmental samples, animals, and humans and were characterized by maximum diversity and represented by different STs.

### BAPS Cluster 2

Bayesian analysis of population structure cluster 2 consisted mostly of ST2 (*n* = 260) and a minor number of other STs. Part of LRSE in the cluster forms a compact group of closely related ST2 genomes from Russia, France, and Germany, all of them harbored a mutation in the 23S rRNA gene (G2576T) and *rpoB* gene (Asp471Glu and Ile527Met). LRSE from France carried an additional mutation in *rpl3* (Met156Thr). Part of LRSE from France and Germany harbored the *cfr* gene. Other LRSE were represented by distantly related ST2 and ST23 genomes from the United States, Brazil, and Germany. A pairwise SNP difference between LRSE and linezolid-susceptible ST2 isolates revealed a low level of identity with a median of 191 SNPs with lower and upper interquartile range (IQR): 181–545. LRSE of ST2 demonstrated high genomic identity with a median pairwise SNP difference of 43 SNPs with lower and upper IQR: 16–53 SNPs. A subgroup of isolates from France, Germany, and Russia demonstrated an even higher level of similarity (Supplementary Table 3). Intragroup SNP differences between genomes from the same country varied from 2 to 27 and intergroup from 44 to 52. BAPS cluster 2 also includes a group of seven highly similar ST23 isolates from the United States with a median pairwise SNP difference of 35 SNPs (IQR: 29–39).

**FIGURE 2:**
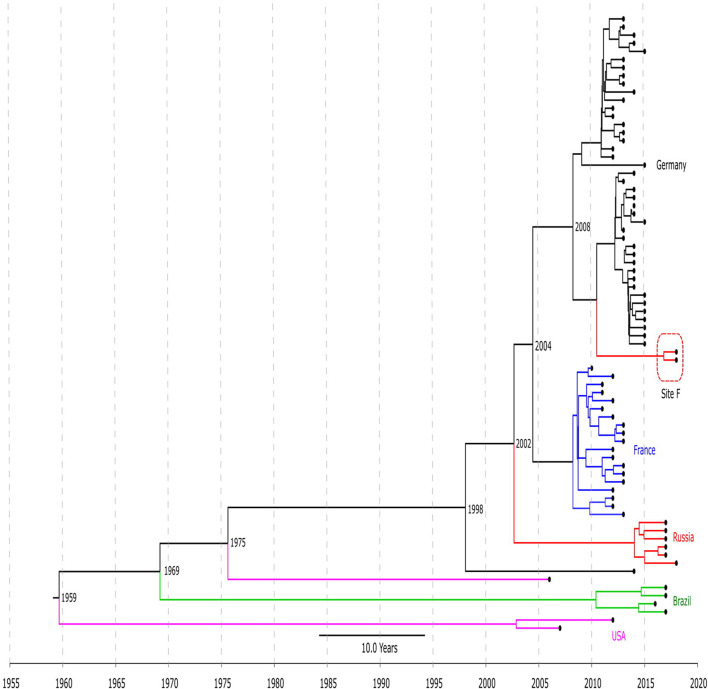
Chronogram of LRSE ST2 (*n* = 76) based on core-SNP alignment and BEAST. Color of branches is matched to country of origin of LRSE: black, Germany; blue, France; green, Brazil, violet, United States; and red, Russia (sites E and F). The time of divergence is given near nodes.

The timed phylogeny analysis of all LRSE ST2 isolates showed that they could have emerged in the 1960s with a large confidence interval: 1915–1994 (Figure 2). A common ancestor of LRSE isolates from Russia, France, and Germany appeared in 1998 (1986–2006) before introduction of linezolid in practice. We can assume two scenarios for the appearance of LRSE in Russia: independent formation (site E) and importation (site F) from Germany. At the same time, the progenitor of the Russian isolates appeared in 2002 (1996–2008). In Brazil and the United States, LRSE isolates emerged independently in 1960–1970.

### BAPS Cluster 3

Bayesian analysis of population structure cluster 3 included ST5, ST2, ST22, ST23, ST186, ST7, ST16, and ST35. Two groups of LRSE were detected within this cluster. The first group included LRSE of ST22 and its single-locus variant ST186 from Russia, France, and the United States. ST22 from Russia and France carried two mutations in 23S rRNA (C2534T and T2504A), and two mutations in *rpl3* (Asp159Tyr and Gly152Asp). Part of the Russian isolates carried a mutation of the *rpoB* gene (His481Asn). ST186 from the United States carried C2534T mutations and harbored the *cfr* gene. The pairwise SNP difference between LRSE and linezolid-susceptible ST22 isolates demonstrated a low level of identity with a median pairwise SNP difference of 214.5 (IQR: 204–233). LRSE from Russia and France were highly similar with a median pairwise SNP difference of 40 (IQR: 35.75–48.25). LRSE of ST186 from the United States were genetically distant from Russian and French isolates with a median pairwise SNP difference of more than 1,000 (Supplementary Table 3).

**FIGURE 3:**
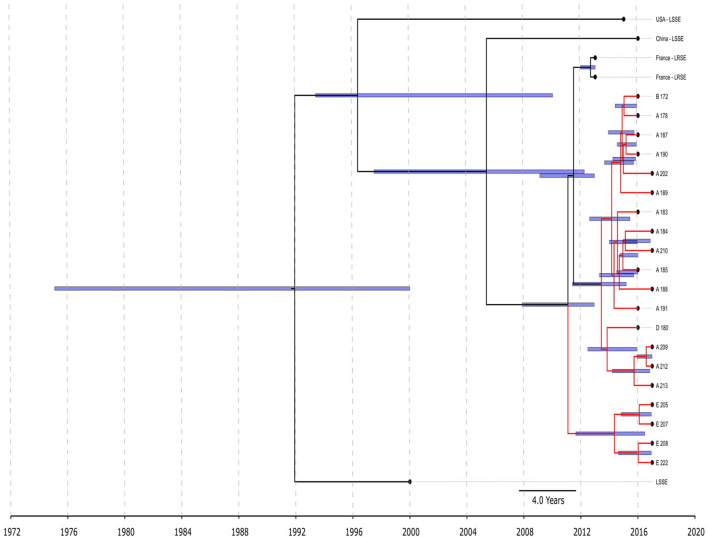
Chronogram of *S. epidermidis* ST22 (*n* = 25) based on core-SNP alignment and BEAST. Three linezolid-susceptible *S. epidermidis* from different countries were included in the analysis. LRSE ST22 from Russia (red branches) and France were included in the analysis. Title of Russian isolates is matched of site isolation. 95% CI of time appearance (blue bars) shown near the nodes.

All ST22 isolates were included in BEAST analysis (Figure 3), a majority of them were LRSE, and a few were susceptible to linezolid. ST22 has a common time of origin in 1992 (1975–2000), but the LRSE sublineage widespread in France and Russia emerged in 2011 (2008–2013), and further divergence continued. Russian isolates from center A, B, and D are descended from a common ancestor with isolates from France, whose time of origin was 2011 (2009–2013). All isolates are compactly localized and have a short spreading period (which is also reflected in short branches on the chronogram), which indicates a clonal spread. Russian isolates from site E formed a separate cluster and were susceptible to rifampicin due to a wild type of *rpoB*.

The second group of LRSE within BAPS cluster 3 consisted of ST5 isolates from France (*n* = 23), and the United States (*n* = 2), and ST23 isolates from the United States (*n* = 2) and Russia (*n* = 2). LRSE of ST23 harbored mutations in 23S RNA (G2576T or C2534T) and *rpoB* (Asp471Glu and Ile527Met), and one isolate carried the *cfr* gene. LRSE of ST5 carried a C2534T mutation and harbored the *cfr* gene. LRSE of ST23 were characterized by significant heterogeneity between BAPS clusters and between all LRSE of ST23 with median pairwise SNP difference of 4,204 (IQR: 4,167–4,252) SNPs and 4,196 (IQR: 37–4,245), respectively. LRSE of ST5 from France were highly similar with a median pairwise SNP difference of 5 (IQR: 2–7) SNP. Two isolates from Russia were also similar, but four isolates from the United States of ST23 and ST5, belonging to BAPS 3 and located close to the Russian isolates, revealed a high level of heterogeneity with a median pairwise SNP difference of 7,165 (IQR: 1,955.5–7,167.5).

In two genomes from Russia (CNS243, CNS244 from site F) and one from the United States (strain DAR4891, BioProject PRJNA308322) belonging to the ST2 rare mutation in *rpl*3, Gly137Val was detected together with a G2576T mutation in 23S rRNA. The role of this SNP in linezolid resistance development is unknown. Mutations in *rpl*2 (Val112Ile and Ile75Thr) and *rpl4* (Asn158Ser) were detected in LRSE and linezolid-susceptible isolates from epidemic sequence types from BAPS cluster 3.

Analysis of distribution of acquired resistance genes in the population shows that the highest mean count of determinants per genome was in genomes of BAPS clusters 2 (6.1) and 3 (5.9). The mean count of resistance genes in the LRSE subpopulation was 6.9.

### Phylogenetic Analysis of *S. hominis*

**FIGURE 4:**
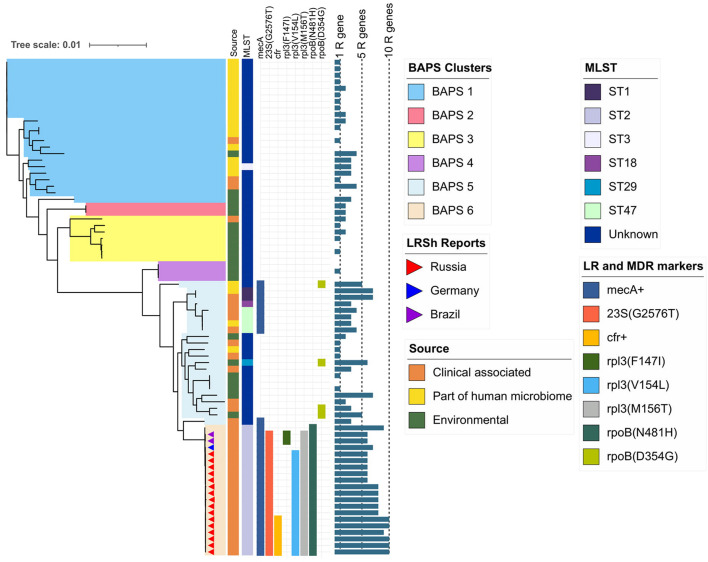
Maximum-likelihood phylogeny of *S. hominis* population (76 genomes) based on core-SNP alignment. Background color fill is matched to BAPS clustering (BAPS 1 to BAPS 6). Linezolid-resistant *S. hominis* isolates (LRSh reports) marked as triangles on the tree. Annotation included source of genomes, MLST, linezolid resistance markers (LR): 23S rRNA mutation G2576T, rpl3 (Phe147Ile, Val154Leu, and Met156Thr), *cfr*; and multidrug-resistance markers (MDR): *mecA* and mutations in *rpoB* (Asn481His, Asp354Gly); bar chart is matched to the number of acquired resistance genes from 0 to 10 genes per genome (1R gene to 10R genes). The following genes were screened (mutations were not included): *aac(6′)-aph(2″), aadD, ant(6)-Ia, ant(9)-Ia, aph(3′)-III, blaZ, cat, ermC, fexA, cfr, fosB, fosD, fusB, fusC, lnu(A), lsa(B), mecA, mph(C), msr(A), str, tetK, tetL, vgaA, and vgaB.*

The pan-genome of *S. hominis* consisted of 7,798 genes, and the core genome included 1,185 genes. Phylogenetic analysis of genomes was based on extraction of 50,332 nt long core genome after alignment. The *S. hominis* population formed six BAPS clusters (Figure 4). The 1–3 BAPS clusters were localized closely to the root; they included *mecA*-negative isolates from healthy humans, animals, insects, and environmental specimens with different new unregistered MLST allelic profiles. Comparative analysis of genomes of these clusters showed a high number of core SNPs: for the BAPS 1 cluster, the pairwise median SNP difference was 4,197 (IQR: 3,538.5–4,915), and for BAPS 3, 2,860 SNPs (IQR: 348–7,694). The pairwise median SNP difference of all BAPS clusters is presented in Supplementary Table 3.

**FIGURE 5:**
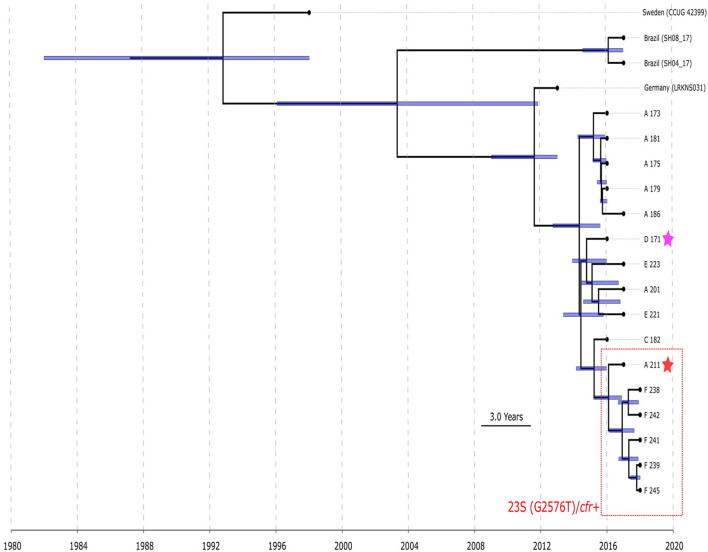
Chronogram of *S. hominis* ST2 (BAPS cluster 6, *n* = 20) based on core-SNP alignment and BEAST. The violet star marks first time isolated oxazolidinone-resistant *S. hominis* in Russia, red star—first isolated cfr-positive *S. hominis*. Sites of appearance oxazolidinone-resistant *S. hominis* is shown on the figure (A, C–F). In addition to Russian isolates, this cluster included (blue names of strains): isolate LRKNS031 (linezolid-resistant *S. hominis* from Germany, BioProject PRJNA314440), two isolates from Brazil (BioProject PRJNA419707 and BioProject PRJNA419709) and linezolid-susceptible isolate CCUG 42399 from Sweden. 95% CI of time appearance (blue bars) shown near the nodes.

BAPS clusters 4–6 consisted of ST1, ST2, ST18, ST29, and ST47. Isolates of BAPS cluster 6 belonged to ST2, and they demonstrated high level genome identity with median 25.5 core SNP differences (IQR: 15–92); the cluster included linezolid-resistant isolates: 16 from Russia (current study), one from Lübeck, Germany (LRKNS031 unpublished, data from BioProject PRJNA314440), and two from Brazil (unpublished, data from BioProjects PRJNA419707 and PRJNA419709). One isolate from Sweden in BAPS cluster 6 was linezolid susceptible. All isolates carried a mutation in *rpoB* (Asn481His). Phylogenetic analysis of a midpoint rooted tree of BAPS cluster 6 (Figure 5) revealed the genetic proximity of the Russian and German isolates; they carried identical mutations in *23S rRNA* (G2603T) and *rpl3* (Met156Thr, Val154Leu). Six Russian isolates carried *cfr* also; most of them were isolated from site F. Isolates with double mechanism resistance and isolates with only mutation in 23S rRNA shared the same core genetic background with a minimum SNP difference. Brazilian isolates were phylogenetically distant; they carried identical mutations in *23S rRNA* but different mutations in *rpl3* (Met156Thr, Phe147Leu). The timed phylogeny analysis (Figure 5) showed that the BAPS 6 cluster of the *S. hominis* ST2 appeared in 1993 (95% CI: 1982–1998). The appearance of the common progenitor of linezolid-resistant *S. hominis* dates to 2003 (1996–2012) soon after introduction of linezolid into clinical practice in 2001, emergence of resistance *de novo* looks more probable.

### Phylogenetic Analysis of *S. haemolyticus*

**FIGURE 6:**
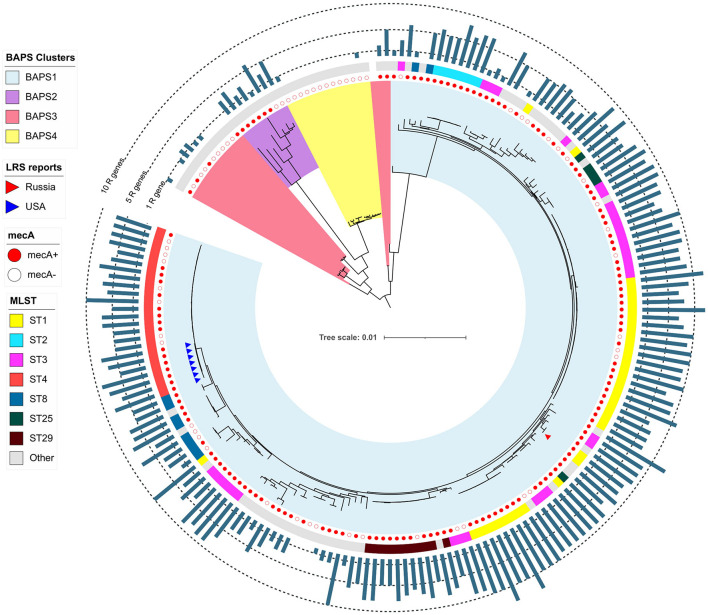
Maximum-likelihood phylogeny of *S. haemolyticus* population (*n* = 207) based on core-SNP alignment. Background color fill is matched to BAPS clustering (BAPS 1 to BAPS 4). Linezolid-restant isolates (LRS reports) isolates is marked triangles: from Russia (one isolate in current study) and the United States (Tewhey et al., 2014). Annotation from inner to outer circle: presence *mecA*; MLST data; outer bar chart is matched to the number of acquired resistance genes (from 0 to 13 genes, specific chromosomal mutations were not included). The following genes were screened: *aac(6′)-aph(2″), aadD, ant(6)-Ia, ant(9)-Ia, aph(3′)-III, blaZ, cat, dfrG, ermA, ermC, cfr, fexA, fosB, fosD, fusB, fusC, lnu(A), lsa(B), mecA, mph(C), msr(A), str, tetC, tetK, tetL, tetM, vgaA, and vgaB.*

The pan-genome of *S. haemolyticus* consists of 13,524 genes. The population of *S. haemolyticus* was divided into four BAPS clusters based on the extraction of 45,692 core SNPs after alignment of 1,032 core genes (Figure 6). BAPS cluster 1 included 82.6% of available *S. haemolyticus* isolates that were recovered at different times from different sources and belonged to 15 different STs, the cluster demonstrated a relatively low-level genome identity with median core-SNP differences of 651 SNPs (IQR: 481–806). A majority (77%) of isolates were *mecA*-positive and carried an average of 6.7 resistant genes per genome. Oxazolidinone-resistant *S. haemolyticus* isolates from Moscow (ST1) and the United States (ST4) (Tewhey et al., 2014) belonged to BAPS cluster 1 and were genetically distant.

### *Staphylococcus haemolyticus* and *S. hominis* Plasmids Carrying *cfr* Genes

One *S. haemolyticus* and six *S. hominis* isolates carried *cfr*-harboring plasmids of approximately 38,000 bp size (only *de novo* assembled contigs were studied). BLAST analysis of the plasmid sequence revealed in GenBank several similar plasmids, which formed two clusters (Supplementary Figure 2). The first cluster included plasmids from *S. haemolyticus* (current study, Moscow), MRSA from the United States (Mendes et al., 2008) and Ireland (Shore et al., 2016), *S. cohnii* from China (Chen et al., 2013), and *S. epidermidis* from France (Dortet et al., 2018). The analysis of core genes of these plasmids revealed differences in no more than five SNPs. The second cluster included similar plasmids from *S. hominis*. They differed from the first cluster in no more than 408 SNPs. Plasmids from both clusters share 90% nucleotide identity and harbored replication gene *repUS5*, which was included in incompatibility group 18 (Inc18). The *Cfr*-gene in all considered plasmids was colocated together with the *fexA* gene coding phenicol resistance.

### Decreased Susceptibility to Glycopeptide in Linezolid-Resistant CoNS

**FIGURE 7:**
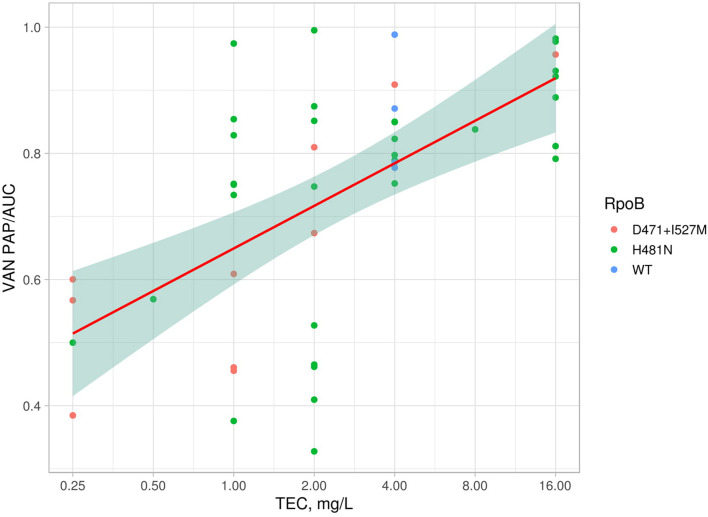
Linear model and confidence interval plot with PAP/AUC data and teicoplanin MICs. Each point represented by AAS in RpoB (WT—wild type).

Eight *S. hominis* ST2 and one *S. epidermidis* ST23 isolates demonstrated teicoplanin resistance with MIC = 16 mg/L. At the same time, the MIC of vancomycin was in range 1–4 mg/L (Table 2). However, the median parameter of AUC_MU50_/AUC_CoNS_ with vancomycin across all isolates was 0.74 (0.38–0.98). Nine isolates demonstrated a hetero-resistant phenotype with AUC_MU50_/AUC_CoNS_ range from 0.90 to 0.99 (Supplementary Table 4). Correlation between susceptibility to rifampicin (including mutations in *rpoB*) and PAP/AUC as well as correlation between teicoplanin and vancomycin levels of susceptibility were not found (Supplementary Figure 3C). However, a moderate positive correlation (*R* = 0.27, *p* < 0.05) was found between two parameters: AUC_MU50_/AUC_CoNS_ and teicoplanin susceptibility (Figure 7). The new lipoglycopeptides (oritavancin, dalbavancin, and telavancin) demonstrated high potency with an MIC range from 0.03 to 0.125 mg/L (Table 2 and Supplementary Table 4). For *S. hominis* isolates, possible genetic markers associated with teicoplanin resistance were identified. These include the plasmid homolog of teicoplanin resistance–related proteins (*tcaA*), localized together with the *cfr* and *fexA*. However, in the *cfr*-positive isolate of *S. haemolyticus* (CNS200), this gene is also present on the plasmid, but the teicoplanin MIC level was 2 mg/L. Other mutations were also identified only in teicoplanin-resistant isolates. In particular, the Tyr75Asn mutation in the protein with unknown function with the Duf420 domain; mutation of Gly95Glu in the protein containing the DedA family protein domain; mutation (G → T) in the upstream region of DNA polymerase III subunit beta.

**TABLE 2:** Glycopeptide and lipoglycopeptides susceptibility and PAP analysis in linezolid-resistant coagulase-negative staphylococci.

	**MLST**	***n***	**Range MIC, mg/L**							**PAP/AUC**		AAS* in RpoB		
			**VAN**	**TEC**	**DLB**	**TLV**	**ORI**	**DAP**	**RIF**	**Range**	M**	**D471E**	**H481N**	**I527M**
LRSE	ST2	8	1–4	0.25–4	0.03–0.06	0.06–0.125	<0.06	0.25–0.5	>4	0.38–0.9	0.59	+	−	+
	ST22	16	1–2	0.125–4	<0.03	0.03–0.125	<0.06	0.125–0.5	0.25–1	0.37–0.99	0.76	−	+	−
	ST22	4	2–4	4	<0.03	0.06–0.125	<0.06	0.25–0.5	<0.03	0.75–0.98	0.85	−	−	−
	ST23	2	2–4	2 and 16	0.06	0.125	0.125	0.5	>4	0.67–0.85	NA	+	−	+
LRSH	ST2	16	1–2	4–16	0.03–0.125	0.06–0.125	0.03–0.125	0.125–0.5	0.5–2	0.40–0.9	0.75	−	+	−

NA, not applicable; VAN, vancomycin; TEC, teicoplanin; DLB, dalbavancin; TLV, telavancin; ORI, oritavancin; DAP, daptomycin; RIF, rifampicin. *amino acid substitution, **median.

Using genomic data of all isolates included in the study, we analyzed the possible association of linezolid-resistance in *S. epidermidis* and *S. hominis* isolates with a decrease susceptibility to glycopeptides. For this purpose, 17 amino acid sequences of homologous proteins involved in the decreased susceptibility to glycopeptides in *S. aureus* were analyzed. A total of 45 mutation variants were identified, including missense and frameshift mutations. Using MCA analysis, the distribution of these mutations in the proteins was not associated with the LRSE genomes (Supplementary Figure 3B). However, it was found that the following mutations: YycH (Ser379Ala), RpoB (Ser486Tyr), GraS (Asn2Asp), and GraR (Glu224Gly) are most common in LRSE (*p* < 0.01), then in the other groups (heat map of frequency of occurrence mutations is presented in the Supplementary Figure 3A). There were no significant differences in the prevalence of these mutations between linezolid-resistant and linezolid-susceptible *S. hominis*.

## Discussion

In 2018, the main representative of CoNS group *S. epidermidis* was recognized by the European Centre for Disease Prevention and Control (2018) as a public health threat. The decision was based on the results of a study (Lee et al., 2018). The authors described the international spread of three hospital-adapted, multidrug-resistant lineages of *S. epidermidis.* Among the multidrug-resistant *S. epidermidis* isolates included in the study, the 18 isolates from Germany, France, and Ireland demonstrated resistance to linezolid, 14 of them belonged to ST2, three to ST5, and one to ST23. In the current study, we examined the international spread of LRSE in more detail. We also analyzed the dissemination of linezolid-resistant lineages of other common human skin colonizers: *S. hominis* and *S. haemolyticus.*

Evaluation of pairwise core-SNP differences between isolates of the same group (intragroup comparison) or between isolates of different groups (intergroup comparison) is a powerful tool for the assessment of the level of similarity between bacteria. However, criteria for different levels of similarity or identity are not established, making it difficult to interpret the results and differentiate lineages that have independently acquired resistance to oxazolidinones from lineages originating from a common resistant precursor. In this case, additional approaches could be used, such as BAPS and BEAST, for more detailed analysis of *Staphylococcus* phylogenomic.

In the current study using several approaches, we uncovered the existence of three international LRSE lineages, which largely coincides with the clustering of the *S. epidermidis* obtained in the study (Lee et al., 2018). The first lineage was represented by ST2 BAPS cluster 2 with highly similar isolates from France, Germany, and Russia harboring identical mutations in 23S rRNA and *rpoB*. The SNP difference between genomes from the same country (intragroup) was less than between genomes from different countries (intergroup). The time-scaled tree analysis showed that a common ancestor for LRSE ST2 from European countries and Russia appeared in 1998 (1986–2006) before introduction of linezolid in clinical practice, which indicates a greater likelihood of independent formation of LRSE in various countries. However, possible import of isolates can be observed for isolates from site F, which are in the same clade with LRSE from Germany. After dissemination to different regions, the lineage probably continues to evolve, thus the sublineage in France acquired additional a mutation in *rpl3*-Met156Thr, and some isolates in Germany and France acquired *cfr* genes.

The second LRSE lineage included ST22 isolates from France and Russia and its single-locus variant ST186 from the United States. Two ST22 isolates from France (Dortet et al., 2018) and 20 from Russia demonstrated high levels of intra- and intergroup similarity; they have identical mutations in 23S rRNA and *rpl3*, which suggests the existence of a common resistant precursor that appeared in 2011 (2008–2013). Isolates of this lineage harbored a maximal number of acquired resistance genes between all studied genomes. This genetic lineage may be common not only in Russia and France. ST22 LRSE carrying the same mutations in 23S and *rpl3* were reported from Greece and Turkey (Karavasilis et al., 2015; Freitas et al., 2018; Papadimitriou-Olivgeris et al., 2020). The whole genome sequencing (WGS) data in the mentioned publication are lacking, and it is impossible to evaluate the level of similarity between ST22 isolates from different sources. Seven ST186 isolates from the United States were genetically distant from the ST22 subgroup.

The third LRSE lineage included highly similar ST5 isolates from France (*n* = 23) and the United States (*n* = 2), *cfr* genes, and the 23S rRNA SNP at position 2,534 mediated oxazolidinone resistance in this subgroup. In this case, neither the international spread of LRSE nor the independent acquisition of the plasmid by representatives of closely related genetic lineages can be ruled out.

*S. hominis* is a poorly studied species among CoNS, and it is an uncommon causative agent of different opportunistic infections, including life-threatening nosocomial (d’Azevedo et al., 2008) and neonatal bacteremia’s (Chaves et al., 2005). ST16, ST23, and ST2 are major lineages associated with human infections (Zhang et al., 2013). In this study, we found emergence and spread of linezolid-resistant ST2 lineage in Russia, Germany, and Brazil. BEAST analysis revealed that time of divergence of linezolid-resistant *S. hominis* was 2003 (1996–2012). The greatest genetic relationship with isolates from Russia demonstrated a single isolate from Germany. Linezolid-resistant *S. hominis* were previously reported from Europe (Musumeci et al., 2016; Drăgulescu et al., 2018) and Brazil (de Almeida et al., 2013; Chamon et al., 2014); however, data on MLST typing and/or WGS of these isolates are lacking. Six isolates from Russia carried simultaneous mutations in genes 23S rRNA and plasmid-born *cfr*. This combination of resistance mechanisms was previously described only in Romania (Drăgulescu et al., 2018). It cannot be ruled out that the spread of linezolid-resistant *S. hominis* ST2 is underestimated.

Several linezolid-resistant *S. hominis* from Russia demonstrated resistance to teicoplanin while maintaining susceptibility to vancomycin. To our knowledge, only a few reports dealing with teicoplanin-resistant *S. hominis* are published (Cercenado et al., 1996; d’Azevedo et al., 2008). We propose that resistance is caused by mutations in hypothetical proteins with Duf420 and DedA domains. The DedA family membrane proteins are widely represented in Gram-negative and Gram-positive bacteria; however, their biological functions are unknown. In one study, it was shown that DedA protein is associated with colistin resistance in *Burkholderia* (Panta et al., 2019). Further studies are needed for understanding of glycopeptide-resistance mechanisms in CoNS.

*Staphylococcus haemolyticus* is also an opportunistic pathogen and the second most frequent CoNS isolated from human blood cultures. In the study of Cavanagh et al. (2014), the population structure based on analysis of the core-genomes of a large collection of clinical European *S. haemolyticus* isolates showed predominance of one single cluster of genomes. All genomes from Cavanagh’s study were included in the current work, and a majority of them were in BAPS cluster 1. This cluster included highly similar linezolid-resistant isolates from the United States (Tewhey et al., 2014) and genetically distant isolate from Russia. Linezolid-resistant *S. haemolyticus* were previously reported from Europe (Rodriguez-Aranda et al., 2009), China (Jian et al., 2018), and India (Brijwal et al., 2016; Mittal et al., 2019); however, data on MLST typing and\or WGS of these isolates are lacking.

A limitation of the study is the impossibility to characterize mobilomes from the short reads of the studied genomes. We were able to demonstrate that *cfr* harboring plasmids from *S. hominis* and *S. haemolyticus* belonged to different clusters of *repUS5*-like plasmids widely disseminated in the *S. aureus* population (Mendes et al., 2008; Chen et al., 2013; Dortet et al., 2018). Lack of epidemiological data supporting this assumption is another limitation of the study. We also have no information about the level of consumption of antibiotics, including linezolid in participating hospitals, which may indicate in favor of the local formation of resistance.

Noteworthy is the small number of available complete genomes of linezolid-resistant strains of CoNS that are not associated with the main genetic lineages. Many linezolid-resistant clones may be quickly eliminated from circulation, and only evolutionarily successful ones remain. However, likely, isolates obtained from local outbreaks were mainly included in the studies with genome-wide sequencing. Larger studies using whole genome sequencing are needed to better understand the molecular epidemiology of linezolid-resistant CoNS.

## Conclusion

CoNS are part of the human microbiome and are frequent contaminants of implants and medical devices. The importance of CoNS in the future is likely to increase as the use of invasive technologies in medicine increases, which will require new approaches to antibiotic therapy and, possibly, wider use of oxazolidinones. At present, the global population of linezolid-resistant CoNS is represented by a limited number of homogeneous genetic lineages and a small number of unrelated isolates. The leading mechanisms of resistance are mutations in the 23S rRNA and ribosomal protein genes; resistance due to *cfr* production is relatively rare. The geographic dissemination of resistance to linezolid is mediated by both the spread of resistant clones (LRSE ST22) and the formation of resistance *de novo* in closely related lineages of (LRSE ST2 and *S. hominis* ST2). The rate of further dissemination of resistance in the future is likely to depend on the consumption of oxazolidinones; however, it is almost impossible to predict which of the resistance mechanisms will dominate. Whole genome sequencing should become the main tool in the surveillance of the spread of linezolid-resistant CoNS.

## Data Availability Statement

Genomic data have been deposited in NCBI Sequence Read Archive (SRA) and all reads are available from BioProject PRJNA384130 (SRA id: SRR5482186–SRR5482205 and SRR8427123–SRR8427149).

## Author Contributions

SS, VG, and AK conceived and designed the study. SL and DL analyzed the data. OK, MB, ND, ZG, TP, LL, and AG performed the experiments. All authors have read and agreed to the published version of the manuscript.

## Conflict of Interest

The authors declare that the research was conducted in the absence of any commercial or financial relationships that could be construed as a potential conflict of interest.

## Publisher’s Note

All claims expressed in this article are solely those of the authors and do not necessarily represent those of their affiliated organizations, or those of the publisher, the editors and the reviewers. Any product that may be evaluated in this article, or claim that may be made by its manufacturer, is not guaranteed or endorsed by the publisher.

## Footnotes

1
http://www.eucast.org

2
https://github.com/tseemann/mlst

3
https://github.com/tseemann/abricate

4
https://github.com/sleyn/panmap

5
https://github.com/MDU-PHL/pairwise_snp_differences

6
https://github.com/tseemann/barrnap

7
https://github.com/dariader/Riddikulus

## Supplementary Material

Supplementary Figure 1 Maximum-likelihood phylogeny of *S. epidermidis* population based on core-SNP alignment with additional data and names of strains and description of calculated acquired resistance genes.

Supplementary Figure 2 Structure alignment of *cfr*-harboring plasmids.

Supplementary Figure 3 **(A)** Heat map of the distribution of AAS in proteins associated with decreased susceptibility to glycopeptides in *S. epidermidis*; **(B)** Results of MCA analysis; **(C)** Distribution of isolates with PAP data, genotypes, and variants of mutations in RpoB.

Supplementary Table 1 Quality of sequence data.

Supplementary Table 2 Genomes included in the study.

Supplementary Table 3 Pairwise SNP differences between genomes of CoNS.

Supplementary Table 4 Description of resistance phenotypes and genotypes of Russian linezolid resistant isolates including MICs and PAP data.
