# Supplementary material for: Global Expansion of Linezolid-Resistant Coagulase-Negative Staphylococci
Source: Front Microbiol. 2021 Sep 13;12:661798. doi: 10.3389/fmicb.2021.661798 (PMC8473885; doi:10.3389/fmicb.2021.661798)
Supplement: Supplementary Figure 1 — Maximum-likelihood phylogeny of S. epidermidis population based on core-SNP alignment with additional data and names of strains and description of calculated acquired resistance genes. [file Data_Sheet_1.PDF]

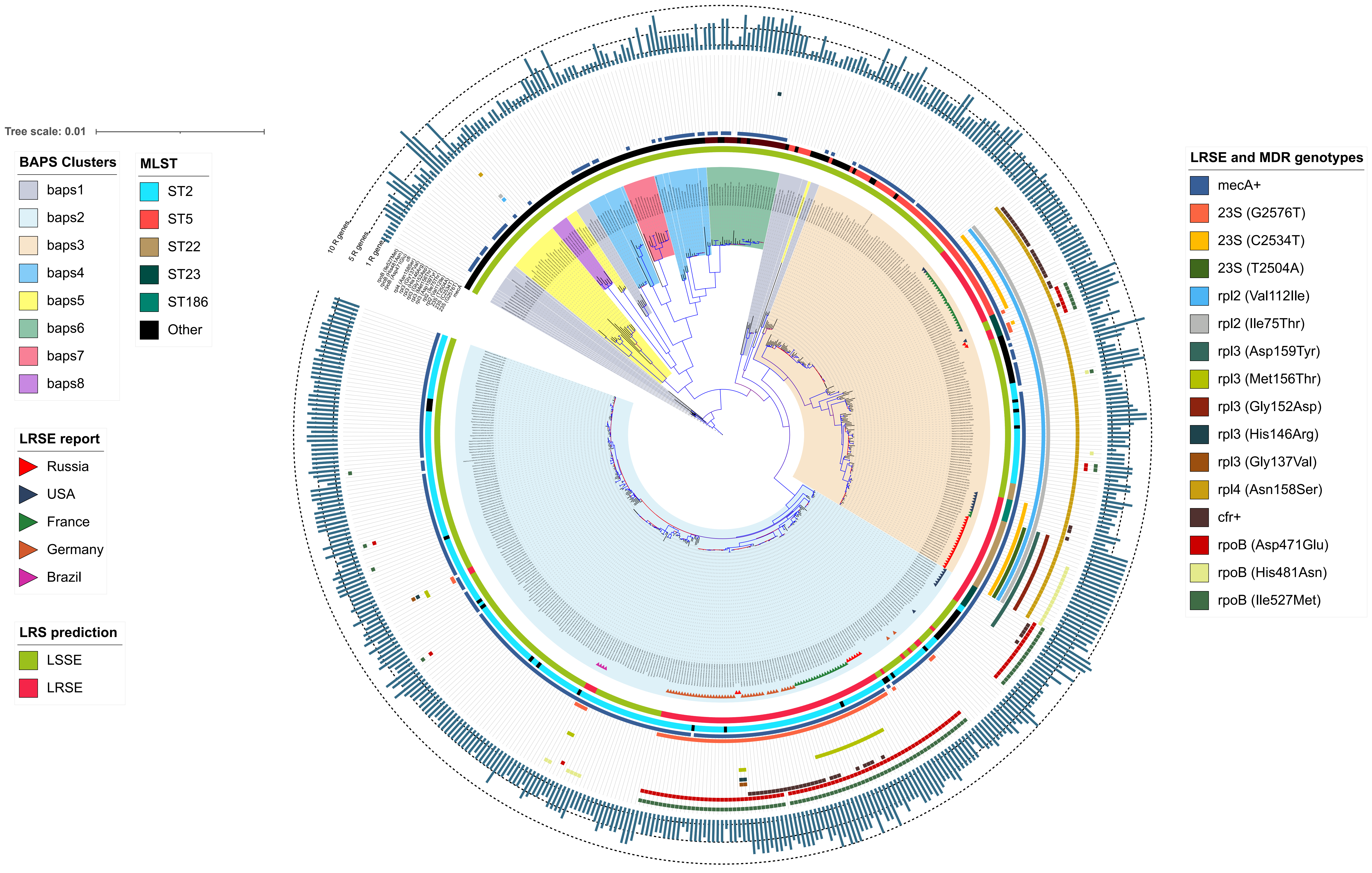

Tree scale: 0.01
BAPS Clusters
baps1
baps2
baps3
baps4
baps5
baps6
baps7
baps8
MLST
ST2
ST5
ST22
ST23
ST186
Other
LRSE report
Russia
USA
France
Germany
Brazil
LRS prediction
LSSE
LRSE
10 R genes
5 R genes
1 R genes
LRSE and MDR genotypes
mecA+
23S (G2576T)
23S (C2534T)
23S (T2504A)
rpl2 (Val112Ile)
rpl2 (Ile75Thr)
rpl3 (Asp159Tyr)
rpl3 (Met156Thr)
rpl3 (Gly152Asp)
rpl3 (His146Arg)
rpl3 (Gly137Val)
rpl4 (Asn158Ser)
cfr+
rpoB (Asp471Glu)
rpoB (His481Asn)
rpoB (Ile527Met)

Supplemental material Fig. S1. Maximum-likelihood phylogeny of *S. epidermidis* population (n=554) based on core-SNP alignment. Color of the branches is matched bootstrap analysis scale in range 51 – 100, blue color is matched maximum bootstrap, red – the minimum. Background color fill is matched to BAPS – clustering (BAPS 1 to BAPS 8). LRSE isolates is marked triangles: from Russia (current study), USA (Tewhey et al., 2014), France (Dortet et al., 2018), Germany (unpublished data, only metadata is available BioProject PRJNA314440) and Brazil (unpublished data, only metadata is available from BioProjects PRJNA419710, PRJNA419705, PRJNA419706 and PRJNA419711). Annotation from inner to outer circle: *in silico* linezolid resistance prediction; MLST data (only prevalence STs are shown: ST2, ST5, ST22, ST186, ST23 and ST59); presence *mecA*, linezolid resistance (mutations G2576T, C2534T, T2504A, rpl2 (Val112Ile, Ile75Thr), rpl3 (Asp159Tyr, Met156Thr, Gly152Asp, His146Arg, Gly137Val), rpl4 (Asn158Ser) genes and presence of *cfr*) and rpoB mutations (Asp471Glu, His481Asn, Ile527Met). The outer bar chart is matched to the number of acquired resistance genes (from 1 to 13 genes, specific chromosomal mutations were not included here). Followed genes were screened: *mecA, blaZ, aacA-ACI1, aadA1, ant(4')-Ia, ant(6)-Ia, ant(9)-Ia, aph(2'')-If, aph(2'')-Ih, aph(3')-IIIa, aph(6)-Id, catA7, catA8, cfr, dfrC, dfrG, ermA, ermC, fexA, fosA6, fosB, fosD, fusB, fusC, lnuA, IsaB, mefA, mphC, msrA, msrD, mupA, tetB, tetC, tetK, tetM, vgaA, vgaB*.
