# Supplementary material for: Global Expansion of Linezolid-Resistant Coagulase-Negative Staphylococci
Source: Front Microbiol. 2021 Sep 13;12:661798. doi: 10.3389/fmicb.2021.661798 (PMC8473885; doi:10.3389/fmicb.2021.661798)
Supplement: Supplementary Figure 2 — Structure alignment of cfr-harboring plasmids. [file Data_Sheet_2.PDF]

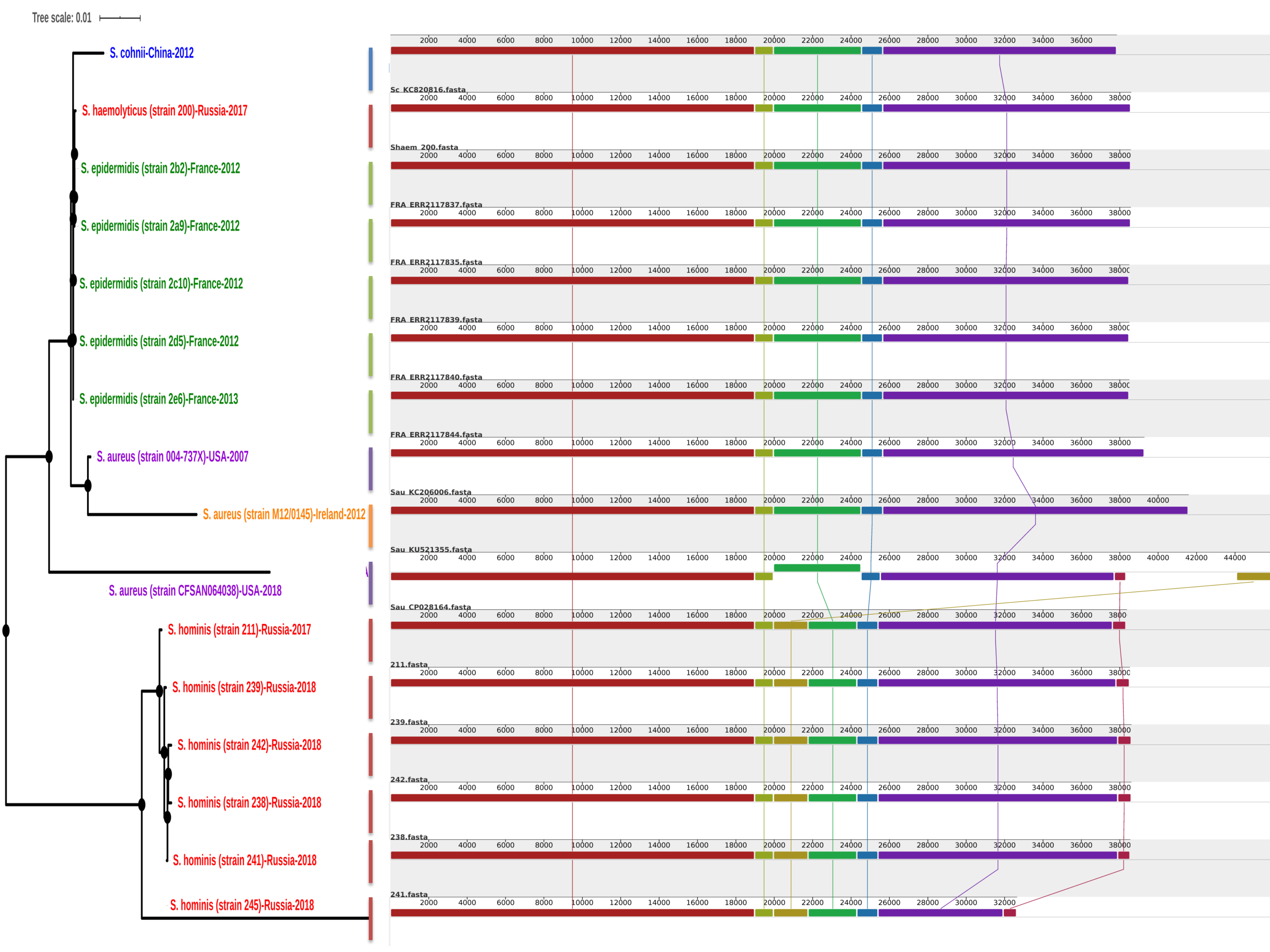

Tree scale: 0.01
S. cohnii-China-2012
S. haemolyticus (strain 200)-Russia-2017
S. epidermidis (strain 2b2)-France-2012
S. epidermidis (strain 2a9)-France-2012
S. epidermidis (strain 2c10)-France-2012
S. epidermidis (strain 2d5)-France-2012
S. epidermidis (strain 2e6)-France-2013
S. aureus (strain 004-737X)-USA-2007
S. aureus (strain M12/0145)-Ireland-2012
S. aureus (strain CFSAN064038)-USA-2018
S. hominis (strain 211)-Russia-2017
S. hominis (strain 239)-Russia-2018
S. hominis (strain 242)-Russia-2018
S. hominis (strain 238)-Russia-2018
S. hominis (strain 241)-Russia-2018
S. hominis (strain 245)-Russia-2018
Sc_KC820816.fasta
Shaem_200.fasta
FRA_ERR2117837.fasta
FRA_ERR2117835.fasta
FRA_ERR2117839.fasta
FRA_ERR2117840.fasta
FRA_ERR2117844.fasta
Sau_KC206006.fasta
Sau_KU521355.fasta
Sau_CP028164.fasta
211.fasta
239.fasta
242.fasta
238.fasta
241.fasta

Supplemental material Figure S2. Structure alignment of *cfr*-harboring plasmids. In analysis were included *S. cohnii* (blue color name, GenBank - KC820816.1) *S. hominis* and *S. haemolyticus* plasmids (red color name, current study); *S. epidermidis* (green color name, GenBank BioProject - PRJEB22222); *S. aureus* strain 004-737X (KC206006.1) and *S. aureus* strain CFSAN064038 (CP028164.1), violet color names; *S. aureus* strain M12/0145 (orange color name, KU521355.1); Year is matched of date isolation. Collinear blocks (regions with high sequence identity without rearrangements) produce by Mauve software shown in alignment with different colors. Phylogenetic tree were calculate using SNPs extracted from plasmids core-genes.
