# Supplementary material for: Global Expansion of Linezolid-Resistant Coagulase-Negative Staphylococci
Source: Front Microbiol. 2021 Sep 13;12:661798. doi: 10.3389/fmicb.2021.661798 (PMC8473885; doi:10.3389/fmicb.2021.661798)
Supplement: Supplementary Figure 3 — (A) Heat map of the distribution of AAS in proteins associated with decreased susceptibility to glycopeptides in S. epidermidis; (B) Results of MCA analysis; (C) Distribution of isolates with PAP data, genotypes, and variants of mutations in RpoB. [file Data_Sheet_3.PDF]

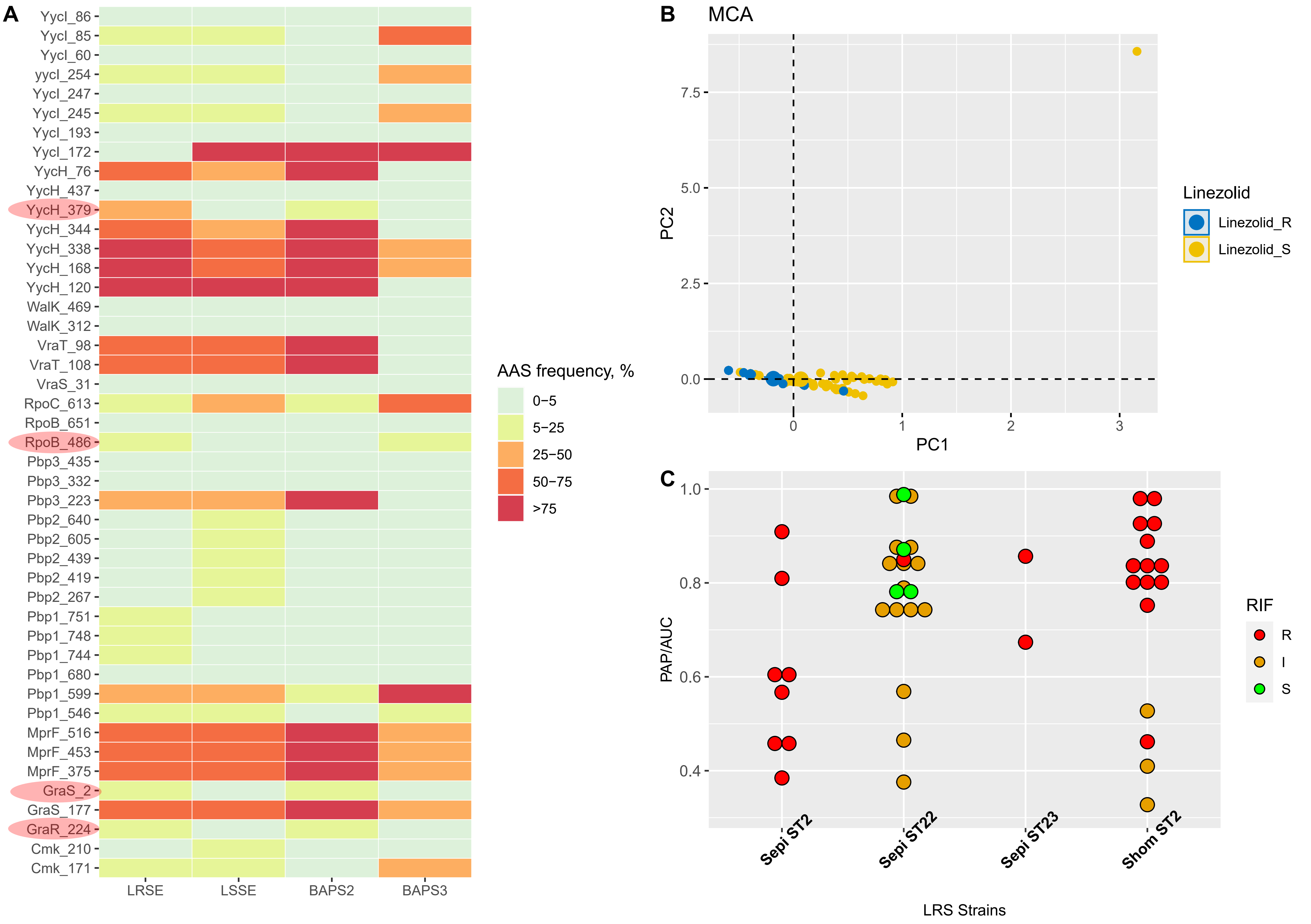
A
YycI_86
YycI_85
YycI_60
yycI_254
YycI_247
YycI_245
YycI_193
YycI_172
YycH_76
YycH_437
YycH_379
YycH_344
YycH_338
YycH_168
YycH_120
WalK_469
WalK_312
VraT_98
VraT_108
VraS_31
RpoC_613
RpoB_651
RpoB_486
Pbp3_435
Pbp3_332
Pbp3_223
Pbp2_640
Pbp2_605
Pbp2_439
Pbp2_419
Pbp2_267
Pbp1_751
Pbp1_748
Pbp1_744
Pbp1_680
Pbp1_599
Pbp1_546
MprF_516
MprF_453
MprF_375
GraS_2
GraS_177
GraR_224
Cmk_210
Cmk_171
LRSE
LSSE
BAPS2
BAPS3
AAS frequency, %
0–5
5–25
25–50
50–75
>75
B
MCA
PC2
PC1
7.5
5.0
2.5
0.0
0
1
2
3
Linezolid
Linezolid_R
Linezolid_S
C
PAP/AUC
1.0
0.8
0.6
0.4
Sepi ST2
Sepi ST22
Sepi ST23
Shom ST2
LRS Strains
RIF
R
I
S

Fig S3. LRSE and analysis of decreased susceptibility to glycopeptides A. Heat map of the distribution of AAS in proteins associated with decreased susceptibility to glycopeptides in *S. epidermidis*. Distribution into four groups - all LRSEs, all LSSEs, BAPS2 and BAPS3 genomes. The names contain the names of the protein, the position with the substitution. Mutations that are associated with the only LRSE group are marked in red. B - Results of MCA analysis. Clusters associated with LRSE or LSSE was not found. C - Distribution of isolates with PAP data, genotypes, and variants of mutations in RpoB. There was no direct relationship between rifampicin resistance and the results of PAP analysis.
